# Recent updates on the biological efficacy of approved drugs and potent synthetic compounds against SARS-CoV-2

**DOI:** 10.1039/d2ra06834f

**Published:** 2023-01-26

**Authors:** Anjani, Sumit Kumar, Brijesh Rathi, Poonam

**Affiliations:** a Department of Chemistry, Chaudhary Charan Singh Haryana Agricultural University Hisar Haryana-125004 India; b Department of Chemistry, Miranda House, University of Delhi Delhi-110007 India poonam.chemistry@mirandahouse.ac.in; c Laboratory for Translational Chemistry and Drug Discovery, Hansraj College, University of Delhi Delhi-110007 India; d Delhi School of Public Health, Institute of Eminence, University of Delhi Delhi-110007 India

## Abstract

The novel severe acute respiratory syndrome coronavirus 2 (SARS-CoV-2), also known as COVID-19, has triggered a global pandemic that has prompted severe public health concerns. Researchers worldwide are continuously trying to find options that could be effective against COVID-19. The main focus of research during the initial phase of the pandemic was to use the already approved drugs as supportive care, and efforts were made to find new therapeutic options. Nirmatrelvir (PF-07321332), a Pfizer chemical, recently received approval for usage in conjunction with ritonavir. This mini-review summarises the biological effectiveness of vital synthetic compounds and FDA-approved medications against SARS-CoV-2. Understanding how functional groups are included in the creation of synthetic compounds could help enhance the biological activity profile of those compounds to increase their efficacy against SARS-CoV-2. This opened the way for researchers to explore opportunities to develop better therapeutics by investigating synthetic analogs.

## Abstract 2

The role of functional group in discovery of Nirmatrelvir is valuable and interesting for development of various inhibitors against viral diseases.

## Graphical abstract

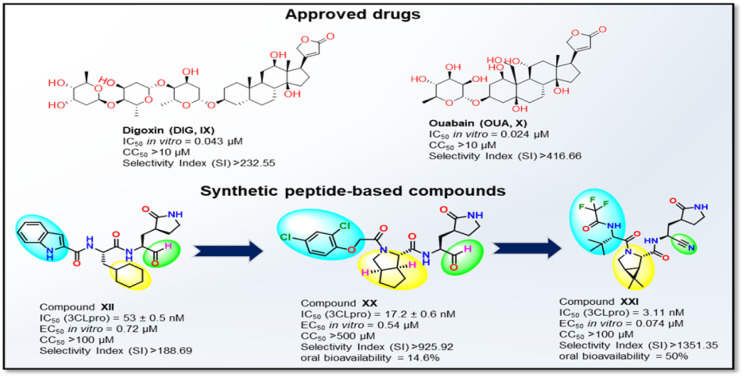

## 1. Introduction

SARS-CoV-2 (severe acute respiratory syndrome coronavirus 2), the seventh human coronavirus, is one of the deadliest virus. As of 25 December 2022, there have been more than 661 million reported infections and nearly 6.68 million reported deaths from COVID-19.^1^ It is a single-stranded RNA beta coronavirus, and its genome encodes non-structural proteins (nsps), structural proteins, and several accessory proteins.^2–4^ Since its outbreak, tremendous efforts have been made to resolve the COVID-19 threat. Although vaccines may be good in ‘preventing’ disease with the emergence of new strains of microbes, their efficacy may reduce and is seen in many countries during the mutation of new strains.^5,6^ Currently, Omicron is the latest strain circulating variants for the virus, which is a matter of concern around the globe. By mid-December, the WHO reported that Omicron had already been discovered in 77 countries worldwide, initially with many cases among tourists returning from Africa.^7^ Around 150 countries have reported Omicron cases, and it is well known that this variant of concern (VOC) is very contagious and has a heightened propensity to infect those who are ill or who have recently had vaccinations.^8,9^ This is supported by multiple recent studies that show the Omicron VOC has a much-decreased susceptibility to neutralizing antibodies produced by prior SARS-CoV-2 infection or vaccination.

Drugs, however, are ideally adapted to “treat” a condition. Only a significant change in the drug's mechanism of action would cause its efficacy to change in the presence of new strains. Additionally, there are few logistical issues with medicines, making them accessible to nations with middle- and low-income levels. Drugs have the most significant benefit over vaccines, including that they can be used to treat different diseases.^10^ The repurposing of drugs usually takes less time than making a new vaccine or drug for a new infection.^11^ During the initial stages of the COVID-19 pandemic, medications approved for other diseases were used as a supportive care.^12,13^

Current research studies are focus on the importance of main protease (Mpro or 3CLpro) for discovering possible therapeutic for SARS-CoV-2. Additionally, among the SARS-CoV-2 mutant strains, nearly no mutations have been found in the Mpro active region, making this enzyme a promising therapeutic target.^14–16^ Also, Mpro is a rational target for anti-SARS-CoV-2 drug development because it only cleaves polypeptide sequences after a glutamine residue.^17–19^ Till now, no human host-cell protease has this substrate specificity, nor does the human genome encode a homogeneic analog to Mpro. His41 and Cys145, the catalytic dyad that promotes the hydrolysis of peptide bonds, represent the center portion of the active site in Mpro, which is surrounded by four pockets that contain essential amino acid residues.^20–22^

The ultimate aim is to develop novel therapeutics designed based on the structure of the virus so that they can fight the mutating strain of the virus. Novel Mpro ligands with various chemical architectures increased target selectivity and enhanced pharmacological profiles must be thoroughly studied and developed in light of the desire to create a wide variety of choices and due to the predicted therapeutic requirements. Multi-site interaction and favorable structure construction techniques were used to combine various segments with the lead structure to fully occupy the active site of Mpro and interact with critical amino acid residues.

Currently, a newly synthesized compound known as Nirmatrelvir (PF-07321332) targeting 3CLpro developed by Pfizer has got approval for its emergency use, and results have shown that the use of Nirmatrelvir in combination with ritonavir, reduced the number of COVID-19 related hospitalizations.^23^ We have reviewed the antiviral data of the synthetic compounds developed exclusively for SARS-CoV-2 along with the biological efficacy of the medications that have been modified and approved for treating other diseases. The main focus among synthesized compounds was on peptide inhibitors as one of them (Nirmatrelvir) was recently approved for it's use against SARS-CoV-2. This mini-review includes the specific data of licensed medications, synthetic peptide-based analogs and some potent non peptidomimetics. This information might be helpful to develop future therapeutic candidates for viral infections.

## 2. Biological efficacy of approved drugs against SARS-CoV-2

**Fig. 1:**
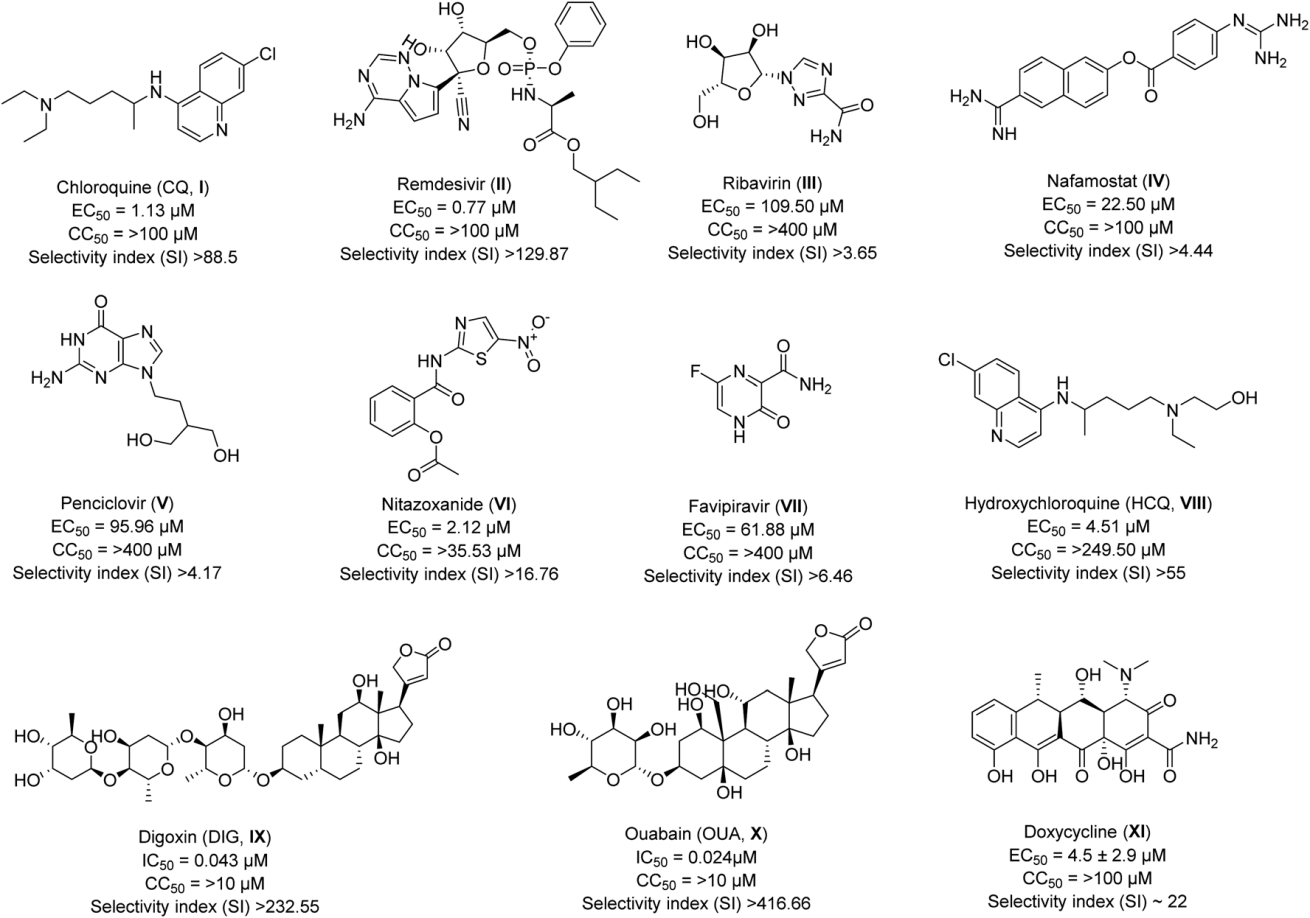
The inhibitory potency in IC_50_ or EC_50_, cytotoxicity, and selectivity index of approved drugs against SARS-CoV-2.

Drug repurposing was the sole method available in the early stages of COVID-19 to combat SARS-CoV-2. Due to the lack of specific therapeutic options, many FDA-approved or currently undergoing clinical trials medications were administered as supportive care. Numerous antivirals and other legal drugs have been examined by researchers and medical professionals all around the world to determine their effectiveness towards SARS-CoV-2. Xiao *et al.*^24^ have evaluated the efficiency of some of the approved drugs and broad-spectrum antivirals against a clinically isolated SARS-CoV-2 strain. They have included drugs like chloroquine (CQ, I), remdesivir (II), ribavirin (III), nafamostat (IV), penciclovir (V), nitazoxanide (VI), favipiravir (VII) for their study. Interestingly, two drugs CQ and remdesivir, have shown activity in the low micromolar range (<5 μM) with 50% maximal effective concentration (EC_50_) values of 1.13 μM and 0.77 μM respectively, as compared to other tested compounds. The cytotoxicity of all the drugs considered for the study was analyzed using CCK8 assay in Vero E6 cells, and 50% cytotoxic concentration (CC_50_) values were found to be >100 μM. Thus, taking the selectivity index (SI) value of drugs CQ and remdesivir is very high, 88.5 and 129.87, respectively (Fig. 1). The other drug which showed SI >10 from selected drugs is nitazoxanide (>16.76; Fig. 1), but it seems to have a cytotoxicity issue with a CC_50_ value of only 35.53 μM requiring *in vivo* evaluation for the drug. Further, the authors have tested the potent hits CQ and remdesivir and calculated their EC_90_ values to be 6.90 and 1.76 μM, respectively.^24^ Overall, *in vitro* results have suggested the high potency of CQ (I) and remdesivir (II) against SARS-CoV-2 infection. Both CQ (I) and remdesivir (II) are known to have safety profiles against Ebola virus^25^ and malaria,^26^ respectively, and thus were recommended to be assessed in human patients suffering from the infection.

There was speculation about the higher antiviral activity of a less cytotoxic derivative of CQ, hydroxychloroquine (HCQ, VIII) which had displayed about 40% less cytotoxicity than CQ in an animal model.^27^ To end the debate, Wang *et al.*^28^ have evaluated the *in vitro* antiviral efficacy of HCQ against SARS-CoV-2 infection, and the results were compared with CQ. The authors have reported the CC_50_ values of 273.20 and 249.50 μM for CQ and HCQ, respectively, in Vero E6 cells. The cytotoxicity evaluation of both drugs did not show any significant difference. For better comparison, the antiviral activity against SARS-CoV-2 for both the drugs was determined at four different multiplicities of infection (MOIs). The results of which showed that CQ has inhibited the SARS-CoV-2 infection more predominately as compared to HCQ as EC_50s_ value for CQ (2.71, 3.81, 7.14, and 7.36 μM) were better than that of HCQ (4.51, 4.06, 17.31, and 12.96 μM) at all MOIs (0.01, 0.02, 0.2, and 0.8) respectively. However, the EC_50_ value of CQ was slightly higher than the previously reported value of 1.13 μM at MOI of 0.05 (Fig. 1) by the same group.^24^ The reason, the authors mentioned was due to the adaptation of the virus in cell culture which led to a significant increase in viral infectivity upon continuous passaging. Overall, they concluded that HCQ (VIII) with SI >55 had the potential to inhibit SARS-CoV-2 infection *in vitro*. However, further validation through *in vivo* and clinical trial results are required to predict the actual potency of HCQ (VIII) to combat COVID-19.

Both drugs (CQ and remdesivir) were used as preventive measures to treat the COVID-19-infected patient during the initial breakdown of the disease. However, on June 15, 2020, FDA (Food and Drug Administration) has revoked the use of CQ (I) as an emergency drug in hospitalized patients due to the associated risk of heart rhythm problems.^29^ Remdesivir (II) continued to be used as supportive care for a brief period, but later, WHO issued a conditional recommendation against the use of remdesivir in hospitalized patients as of November 20, 2020, due to the unavailability of any proof showing improving survival and other outcomes in the patients.^30^ Nevertheless, both drugs have been continuously utilized as positive controls to identify the other hits against SARS-CoV-2.

Ivermectin, an FDA-approved drug known to exhibit potent anti-viral activity against various viruses.^31^ The most effective result displayed by the drug was against the dengue virus (DENV), for which it went for phase III clinical trial. The drug demonstrated a strong safety profile when administered as a single daily oral dose, but no change in viremia (the presence of viruses in the blood) or other clinical benefits were noticed.^32^ Therefore, to see the potency of the drug against the causative agent of the COVID-19 pandemic, Wagstaff *et al.*^33^ explored it for *in vitro* evaluation. The authors have analyzed the Vero cells infected with the Australia/VIC01/2020 strain of SARS-CoV-2 by RT-PCR to replicate SARS-CoV-2 RNA. They observed a reduction of 93% in viral RNA when treated with 5 μM of ivermectin in 24 h, which increased to 99.8% in 48 h and remained consistent at 72 h as compared to the vehicle DMSO. Overall, an ∼5000-fold reduction of viral RNA was noticed in ivermectin-treated compared to control samples by 48 h, and IC_50_ was found to be ∼2 μM.

There were reports regarding the high mortality rate in patients with COVID-19 having problems associated with cardiac diseases than in patients without heart-related issues (51.2% *vs.* 5.1%).^34,35^ Therefore, Choi *et al.*^36^ explored FDA drugs approved for heart diseases, which possess antiviral activity against other coronaviruses. The authors have evaluated the antiviral activity of Digoxin (DIG, IX) and ouabain (OUA, X) against SARS-CoV-2 infection and determined half-maximal inhibitory concentrations (IC_50_). They infected Vero cells with SARS-CoV-2 strain (BetaCoV/Korea/KCDC03/2020) in the presence of selected drugs (DIG and OUA) and positive controls (CQ and remdesivir) at an MOI of 0.01 for 1 h. The IC_50_ values were determined when the nucleocapsid (N) gene was amplified using quantitative real-time PCR (qRT-PCR). The results have indicated the IC_50_ value for DIG (IC_50_ = 0.043 μM) and OUA (IC_50_ = 0.024 μM) were ten-fold better as compared to that of CQ (IC_50_ = 0.526 μM) and remdesivir (IC_50_ = 1.57 μM). Further, cytotoxicity determination was performed using the PrestoBlue cell viability reagent to determine these drugs' SI. The authors have not represented the exact CC_50_ values of the drugs, but the data shown in their report have indicated only 80% and 70% cell survival for DIG and OUA, respectively, at a concentration of 10 μM, with SI  >232.55 and >416.66 (Fig. 1). However, positive controls taken in their study were found to have SI >38.02 and >12.73 for CQ and remdesivir, respectively. The CC_50_ data have shown nearly100% survival of cells for both CQ and remdesivir at 10 μM which remain stable even at 20 μM. In total, both selected drugs, DIG (IX) and OUA (X) displayed potent results during *in vitro* evaluation, but CC_50_ values for both seem to be very low for the recommendation for emergency use, requiring validation for the same through *in vivo* bioassays.

Pradines *et al.*^37^ evaluated Doxycycline (XI), an FDA-approved drug, for its *in vitro* activity against clinically isolated IHUMI-3 (SARS-CoV-2 strain) on Vero E6 cells. The drug is approved as an anti-microbial agent and is used to treat various bacterial infections such as eye infections, urinary tract infections, syphilis, intestinal infections, *etc*.^38^ The drug have shown potent result against the chikungunya virus by blocking entry and replication of the virus at a dose of 11 μM.^39^ It is also reported to block the early-stage replication of another virus, such as respiratory syndrome virus, with a median effective concentration (EC_50_) value of ∼0.5 μM.^40^ That's why authors have selected this particular drug to evaluate its potency against novel coronavirus and compared their results with CQ. The cytotoxicity of both compounds Doxycycline and CQ was determined by assessing Vero E6 cell viability and found to be >100 μM for both. They have reported EC_50_ and EC_90_ (90% effective concentration) values of 4.5 ± 2.9 μM and 23.5 ± 16.5 μM for Doxycycline (Fig. 1), in comparison to 3.2 ± 1.8 μM and 13.9 ± 6.4 μM for CQ (data as per their study). Thus, the selectivity index of the drug was calculated to be ∼22. Overall, their results demonstrated that the drug was compatible with oral uptake (100–200 mg) and intravenous administrations (100 mg) and interacted at both entry and post-entry stages of the SARS-CoV-2 infection. However, no *in vivo* evaluation of the drug molecule in animal model is reported yet to use it as supportive care in case of SARS-CoV-2.

Some repurposed drugs have shown excellent results against SARS-CoV-2 infection during biological assays. But lately, questions have been raised about using some approved drugs, such as CQ and its derivatives, for hospitalized patients. There is always a contradiction between clinical bedside therapy and the academic plausibility of a drug's mechanism of action. With a pandemic looming, it's natural to place high hopes on these medications. It has become apparent over time that it does not give any mortality advantage in hospitalized patients. However, a few studies are being conducted to understand more about these medicines and their involvement in COVID-19. Despite initial snags with toxic effects, history has shown that older medications, such as polymyxins, have come back in clinical use.^41,42^ Only time will execute if these medications will play a role in the fight against SARS-CoV-2 as our understanding of these viruses advances.

## 3. Biological efficacy of peptide based compounds synthesized specifically to combat SARS-CoV-2

Pharmacological discovery includes designing and creating novel drug compounds based on mutation, genomics, and different enzyme interactions. In this regard, peptide-based compounds are appealing chemotypes to have potency against SARS-CoV-2. In the last several decades, peptides with therapeutic promise gained much attention as the number of FDA-approved peptide medications grown prodigiously. They have several advantages, including ease of synthesis, excellent specificity, and low accumulative behavior. Peptide inhibitors have been among the promising anti-Covid medications from many resources since the outbreak of COVID-19. Here, we've highlighted some significant articles about the latest advancements in anti-COVID-19 drug research based on peptide inhibitors.

**Fig. 2:**
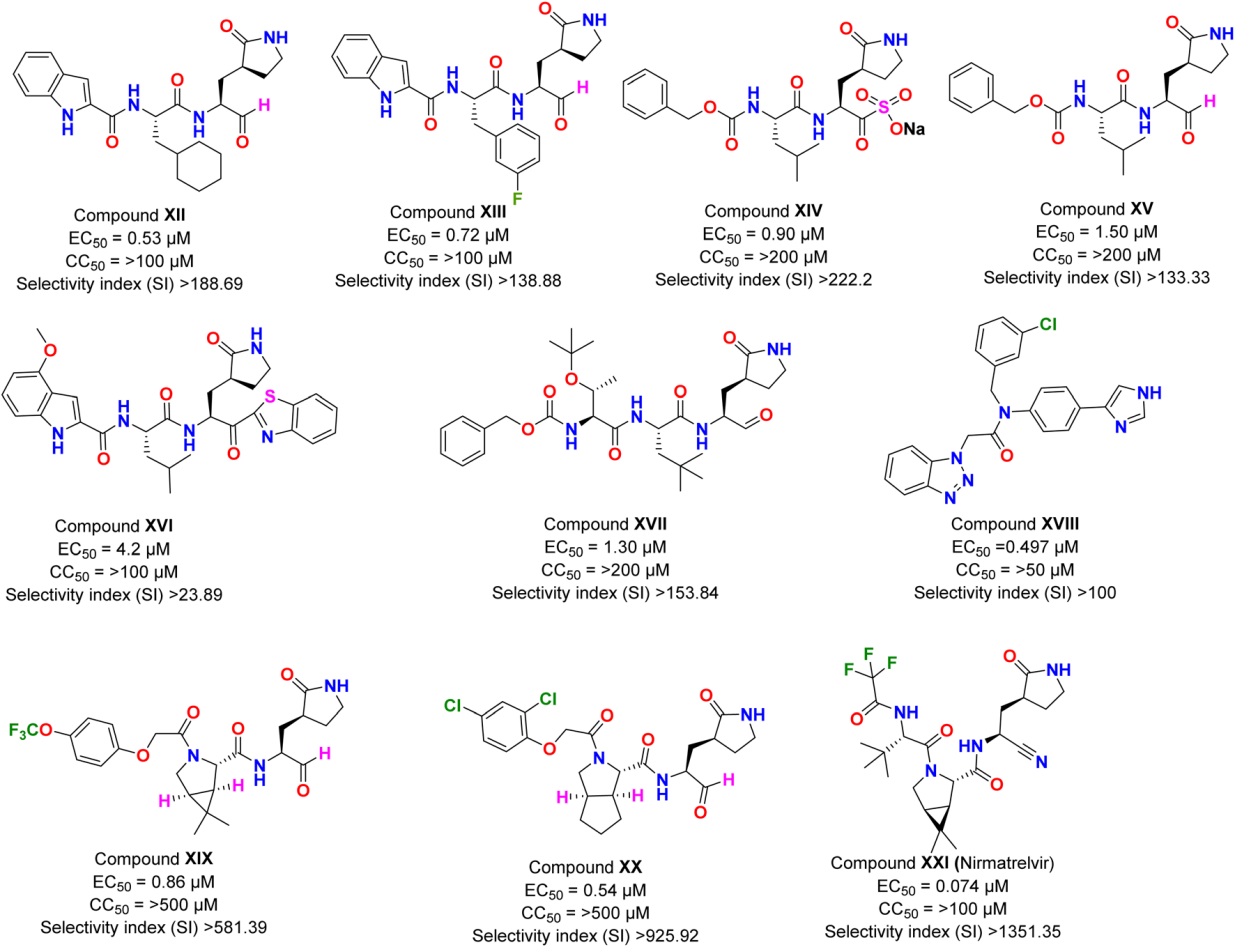
The inhibitory potency in IC_50_ or EC_50_, cytotoxicity, and selectivity index of peptide-based compounds against SARS-CoV-2.

Dai *et al.*^43^ design and synthesize inhibitors targeting SARS-CoV-2 3CLpro by analyzing the substrate-binding pocket. The authors reported two lead compounds with excellent inhibitory activity. In the enzymatic assay, both the compounds (XII and XIII) have shown inhibitory power in the low nanomolar range, with IC_50_ values of 0.053 ± 0.005 μM (XII) and 0.040 ± 0.002 μM (XIII), respectively. Further, these compounds were evaluated for their biological efficacy in cell culture, with XII displaying an EC_50_ value of 0.53 μM with >100 μM CC_50_ (Fig. 2). An almost similar activity profile was shown by compound XIII with an EC_50_ value of 0.72 μM and CC_50_ > 100 μM (Fig. 2). Next, the pharmacological parameters of both compounds were studied in the mice models. The half-life (*t*_1/2_) of the mixture when dosing was done intraperitoneally (5 mg kg^−1^) and intravenously (5 mg kg^−1^), and the microsomal stability assay indicated that compound XII was more effective and displayed good results during *in vivo* pharmacological parameters. These results showed the importance of the cyclohexyl ring over fluoro substituted benzene ring in both the biological efficacy and pharmacological stability of the compounds, as the rest of the chemical composition of both compounds was the same. However, the biological effectiveness of the compound in the mice model was not reported.

Vuong *et al.*^44^ showed that prodrug (XIV) and its parent molecule (XV) were effective inhibitors of the 3CLpro of newly emerged SARS-CoV-2. These two compounds were previously explored for earlier reported coronaviruses, where the prodrug (XIV) inhibited the infection to quite an extent.^45^ In this report, the authors have screened both compounds in enzymatic assay against the 3CLpro enzyme of SARS-CoV-2 and *in vitro* assay against the virus. The prodrug XIV displayed an IC_50_ of 0.19 ± 0.04 μM, whereas the parent compound (XV) showed an IC_50_ value of 0.40 ± 0.05 μM. Both compounds were non-toxic to Vero cells as CC_50_ values for both were >200 μM. The cell-based assay indicated that compound XIV was more effective as compared to its parent compound (XV) with an EC_50_ value of 0.90 μM in comparison to 1.50 μM (Fig. 2). The results from this report have indicated that a slight modification of the structure can bring noticeable changes in the potency of the drug compounds. Here, the probable reason for the prodrug to be more effective is that the sulphonyl salt moiety increased the compound's solubility and improved drug–enzyme interaction. However, the authors have not screened the compound for *in vivo* efficacy against SARS-CoV-2, which could provide more insight into the druggability of the compound.

Konno *et al.*^46^ designed peptidomimetic compounds with a benzothiazole moiety, displaying potent activity against the enzyme 3CLpro of SARS-CoV-2. The most potent inhibitor (XVI; Fig. 2) from their series strongly blocks the SARS-CoV-2 replication with a CC_50_ value of >100 μM and *K*_i_ value = 34.7 ± 2.1 nM. The antiviral assay of the compound revealed that it was completely blocking the SARS-CoV-2 infection at a concentration of 10 μM. However, it's *in vivo* pharmacokinetics and metabolic analyses showed low bioavailability in rats.

Later, Hattori *et al.*^47^ characterized the same compound XVI, earlier explored by Konno *et al.*,^46^ as mentioned above. They discovered that the compound XVI was an irreversible covalent inhibitor of SARS-CoV-2, 3CLpro by X-ray structural analysis with kinetic parameters of *K*_i_ = 2.15 ± 0.49 μM. The antiviral assays for the compounds revealed an EC_50_ value of 4.2 μM.^47^ Overall, such kind of peptidomimetic compounds have shown highly potent results against SARS-CoV-2 infection that could be used as lead for developing therapeutics.

In a recent report by Ma *et al.*,^48^ authors have developed tripeptide-based synthetic inhibitors for 3CLpro enzymes, and their results have shown excellent inhibitory efficacy. The most potent compound (XVII) of the reported series showed EC_50_ value of 1.30 μM (Fig. 2). However, the compound's *in vivo*, and pharmacological data still need to be investigated.

Han *et al.*^49^ synthesized a library of compounds derived from the ML300 series designed for SARS-CoV-1 and used it against the newly emerged coronavirus. Among them, one of the hit compounds showed excellent results. The potent compound (XVIII; Fig. 2) of the series showed the IC_50_ value for the 3CLpro enzyme to be 68 nM, anti-viral inhibition EC_50_ of 0.497 μM, and plaque reduction EC_50_ of 0.56 μM. Overall the compound has shown comparable results to supportive care-approved drugs like remdesivir. However, to comment further on the compound's potential, it is necessary to evaluate its pharmacological parameters along with *in vivo* efficacy against SARS-CoV-2 infection.

Qiao *et al.*^50^ developed a series of 32 compounds based on bicycloproline rings. All compounds were explored for SARS-CoV-2, 3CLpro activity *in vitro*. Two compounds (XIX and XX) showed excellent efficacy in blocking the 3CLpro enzyme of SARS-CoV-2 with IC_50_ values of 15.2 ± 0.4 nM (XIX) and 17.2 ± 0.6 nM (XX). The compounds were also evaluated for their antiviral efficacy against the viral infection, indicating the higher potency of compound XX (EC_50_: 0.54 μM; Fig. 2) over XIX (EC_50_: 0.86 μM; Fig. 2). Next, both compounds were evaluated for their pharmacological parameters and antiviral potential in the mice model. Compound XIX showed an oral bioavailability of 11.2%, and XX displayed it as 14.6% when administered orally. The *in vivo* antiviral assay of both compounds showed that at a single oral dose of 20 mg kg^−1^, compounds could achieve EC_50_. Overall, the hits displayed excellent biological efficacy which could be further optimized to achieve EC_90_ during *in vivo* assays.

Owen *et al.*^51^ discovered an oral available clinical candidate, Nirmatrelvir (XXI) for SARS-CoV-2 infection with a bioavailability of 50% when administered orally. The authors have designed a series of compounds based on previous peptidomimetics analogs discovered by Pfizer. Initially, it was named PF-07321332. The compound was checked for its inhibitory activity against the main protease (3CLpro) enzymes of all human coronavirus, and results have indicated the potent inhibitory activity against all with an IC_50_ value of 3.11 nM against 3CLpro of SARS-CoV-2. The authors have also evaluated the compound against various essential protease present in the human body to study its harmful effect on those proteases, and interestingly compound was not inhibiting any of the vital proteases upto a concentration of 100 μM. Next, the compound was evaluated for its EC_50_ values against the virus in antiviral assays and found to be 0.074 μM with CC_50_ > 100 μM. The pharmacological parameter of the compound was also excellent, making it a perfect candidate for clinical studies. The compound was approved for its first emergency use in the United Kingdom in December 2021 with the combination of ritonavir.^52^ The clinical studies of the compound were conducted and published under trails number NCT04960202. The phase II–III trial results indicated an 89.1% relative risk reduction for the patients.^53^ It was authorized to be used in combination with ritonavir and called Paxlovid by the panel of COVID-19 treatment. Several studies have been conducted on the same compound, such as its stability and robust efficacy in the Syrian hamsters model by Abdelnabi *et al.*^54^ Also the combination assay of the compound was also explored for its synergistic effect.^55^

Nirmatrelvir is given in conjunction with ritonavir, a potent inhibitor of CYP3A enzymes, to prevent its metabolism and raise plasma concentrations of nirmatrelvir. Ritonavir has a sturdy inhibition profile, which increases the risk of drug interactions even though it has therapeutic benefits. Patients and doctors should review the prescribing information for Paxlovid (nirmatrelvir and ritonavir) to assess potential drug interactions with other medications before starting Paxlovid. A cysteine residue in the SARS-CoV-2, 3CLpro is inhibited by nirmatrelvir. The 3CLpro of SARS-CoV-2 and may be other members of the coronavirus family are active because of this cysteine.^56,57^ The major protease, also known as the nsp-5 or 3CLpro, is in charge of cleaving polyproteins 1a and 1ab. The 3CLpro itself, a papain-like cysteine protease, and 14 other nonstructural proteins are all present in these polyproteins.^58^ Nsps, like proteases, cannot be released to carry out their tasks without the 3CLpro's activity, which prevents viral propagation. Till now, no other compound has shown better efficacy than this compound, considering all the parameters such as enzymatic assay, *in vitro*, *in vivo*, oral availability, *etc.*

**Fig. 3:**
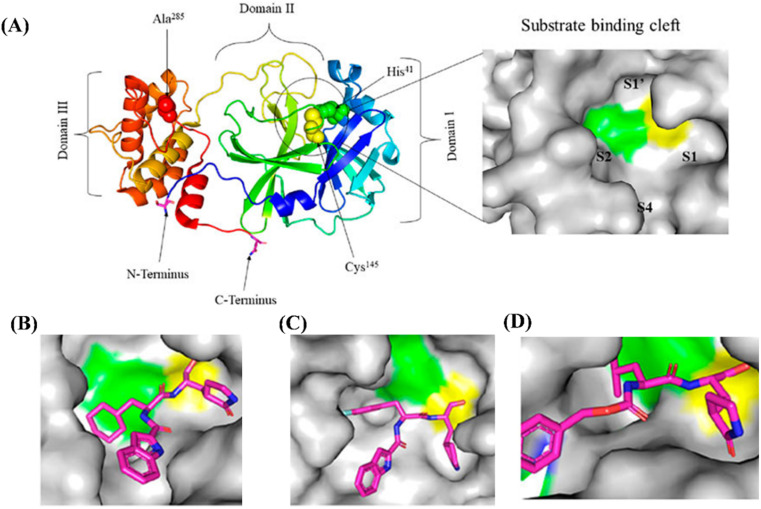
(A) Surface image of the substrate-binding cleft and the crystal structure of free SARS-CoV-2 Mpro solved at 1.75 resolution (PDB entry: 6Y2E). Crystal structure of SARS-CoV-2 Mpro in complex with potential inhibitors. (B) Compound XII (PDB entry: 6LZE, 1.505 Å resolution); (C) compound XIII (PDB entry: 6M0K, 1.504 Å resolution); and (D) compound XIV (PDB entry: 6WTT, 2.15 Å resolution). The figure is adapted with permission from *Front. Chem.*, 12 March 2021, Sec. Medicinal and Pharmaceutical Chemistry, https://doi.org/10.3389/fchem.2021.622898.^59^

The role of functional group on the efficacy of peptide-based synthetic compounds against SARS-CoV-2 can be understood in a better way as shown in Fig. 3A.^59^ In Fig. 3A, there are four subsites in the substrate-binding cleft: S1′, S1, S2, and S4. The amino acids in substrates are numbered P4–P3–P2–P1 and P1′-P2′-P3′ from the N-terminus to the C-terminus of the Mpro, a protein that is conserved among all coronaviruses.^60^ The cleavage site is between P1 and P1′, and the P1 position needs a glutamine residue.^61^ According to research, the most desirable residue for SARS-CoV-2 Mpro to make hydrogen bonds with ligands is Gly143, followed by Glu166, Cys145, and His163.^62^ Because of this, it is crucial to determine the crystal structure of viral proteases in a complex with potential inhibitors because it gives researchers a glimpse into how to improve drugs by tailoring inhibitors to the structural dynamics (monomer or dimer, narrow or wide, deep or shallow) of the target enzyme. Moreover, the dimer of Mpro is the greatest alternative pharmacological target because the monomer is primarily thought to be inactive.^63^ Additionally, designing inhibitors based on how competitively they bind to the active site may aid in finding the most effective inhibitors. Fig. 3B–D provide an explanation of the binding of several compounds (XII, XIII and XIV) with SARS-CoV-2 Mpro.

A cysteine residue in the S1′ subsite of the substrate-binding pocket was covalently anchored from the thiol to sustain the medicines' antiviral action. In the example of XII (Fig. 3B), a C–S covalent bond is formed between the carbon atom of the aldehyde group and Cys145 of the SARS-CoV-2 Mpro. Similar to XII, XIII also exhibits an inhibitory binding mode, with a little variation likely resulting from the downward rotation of the 3-fluorophenyl group of XIII (Fig. 3C). While its (S)-γ-lactam ring at P1 fits in the S1 subsite, the oxygen atom in the aldehyde group in compound XII stabilises the conformation of the medication by creating a hydrogen bond with Cys145's backbone in the S1′ subsite. Therefore, as discussed in the report by Dai *et al.*,^43^ there could be pros and cons of modifying drugs at relevant positions through detailed structural-functional explanations.

**Fig. 4:**
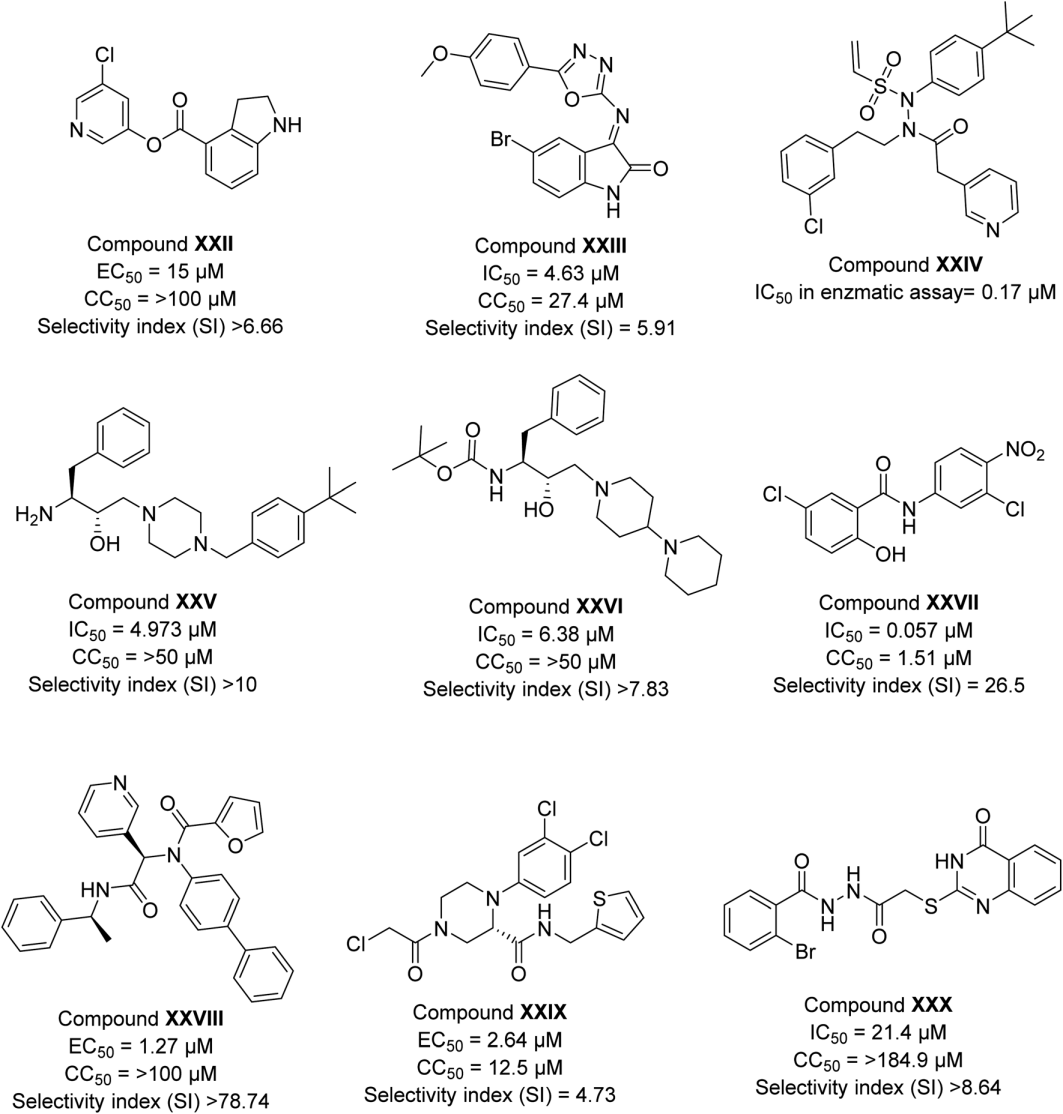
The inhibitory potency in IC_50_ or EC_50_, cytotoxicity, and selectivity index of synthetic compounds against SARS-CoV-2.

Several other reports on the synthetic compounds other than peptidomimetics showed efficacy against SARS-CoV-2 infection. Here, we have compiled the biological data of some potent analogs as depicted in Fig. 4. Hattori *et al.*^47^ explored indoline moiety for the biological efficacy against SARS-CoV-2 infection. The compound (XXII) during antiviral assays revealed an EC_50_ value of 15 μM (Fig. 4). A study by El-Masry *et al.*^64^ showed the *in vitro* activity in the low micromolar range of compounds designed based on 1,3,4-oxadiazoles with the presence of isatin moiety. The most potent compound (XXIII) of their series showed an IC_50_ value of 4.63 μM (Fig. 4). Stille *et al.*^65^ developed some covalent inhibitors of 3CLpro enzymes of SARS-CoV-2 with the introduction of the sulphonyl group and *tert*-butyl group; the designed compound showed a drastic improvement in the inhibition of the enzyme with most compound (XXIV) showed IC_50_ value of 0.17 μM. Kumar *et al.*^66^ have designed a library of a hydroxyethylamine (HEA) based compound that was screened against multiple proteins of SARS-CoV-2, the result of which indicated the strong binding affinity of one of the compounds against nsp15. The compound (XXV) was synthesized by the authors and has been evaluated for biological efficacy against the virus in cell media and depicted excellent results with an IC_50_ value of 4.973 μM. In another report, Gupta *et al.*^67^ followed a similar methodology and suggested the strong binding affinity of three HEA-based compounds against the main protease of SARS-CoV-2. The best result among these was displayed by compound XXVI, an IC_50_ value of 6.38 μM. Juang *et al.*^68^ explored niclosamide analogs based on aniline moiety, and the potent hit (XXVII) of their series exhibited an IC_50_ value of 0.057 μM (Fig. 4) against SARS-CoV-2 infection. In another report by Kitamura *et al.*,^69^ aniline moiety was incorporated in designed analogs; the best hit (XXVIII) of the series showed an efficacy of 1.27 μM.

Recently, Gao *et al.*^70^ investigated the effectiveness of nonpeptidic piperazine compounds against SARS-CoV-2. According to the authors, the top hit (XXIX) of their created series showed a good antiviral potency with an EC_50_ value of 2.64 μM against SARS-CoV-2 infection and an IC_50_ value of 0.18 μM for Mpro. Furthermore, the substance demonstrated more effective target selectivity for SARS-CoV-2 Mpro than for human cysteine proteases. In another recent report, the antiviral activity of two novel series of (tetrahydro)thioquinazoline-*N*-arylacetamides and (tetrahydro)thioquinazoline-*N*-arylacetohydrazides against SARS-CoV-2 was designed, synthesised, and studied by Mohsen and co-workers.^71^ The thioquinazoline-*N*-arylacetamide (XXX) showed better efficacy among all with an antiviral activity (IC_50_) of 21.4 μM (Fig. 4). In the literature, many reports are known for synthetic compounds explored against SARS-CoV-2 infection. Unfortunately, mechanism of action is still nor clear for most of them. Therefore, there is a need to develop advanced biophysical studies to understand the mechanism of action.

## 4. Conclusion

The recent focus of research has been on developing medicines against SARS-CoV-2 infection. There have been numerous attempts to find innovative therapeutic compounds that target different SARS-CoV-2 enzymes. To keep the pipeline full and specifically target novel metabolic pathways crucial for virus survival and spread, it is urgently necessary to create new inhibitors. In the literature, small compounds, peptidomimetics, and FDA-approved medications have demonstrated the ability to interfere with SARS-CoV-2 activity potently. Since one of these compounds received permission for use as an oral medication against SARS-CoV-2 in conjunction with Ritonavir, all peptidomimetics compound with indole moiety, bicyclic rings, aldehyde or ketonic group, and hydrophobic group such as *tert*-butyl have demonstrated higher efficacy. Another interesting observation from all the potent reports related to the antiviral efficacy of the compounds is that 3CLpro was found to be an attractive target to combat the diseases as all the peptidomimetics compounds inhibited the enzyme to quite an extent.

In general, the primary goal of this study is to summarise the recent data regarding the significant synthetic peptide and non-peptide based analogs and approved treatments based on their biological activity against SARS-CoV-2. Importance of functional groups in the drug designing is also highlighted. The information in this study may be used to create synthetic analogs against COVID-19 and investigate potential avenues for creating medicinal therapeutics.

## Abbreviations

SARS-CoV-2: Severe acute respiratory syndrome coronavirus 2
COVID-19: Coronavirus disease in 2019
WHO: World Health Organization
FDA: Food Drug and Administration
CQ: Chloroquine
HCQ: Hydroxychloroquine
MOI: Multiplicities of infection
DIG: Digoxin
OUA: Ouabain
SI: Selectivity Index
IC_50_: 50% inhibitory concentration
CC_50_: 50% cytotoxic concentration
EC_50_: 50% effective concentration
HEA: Hydroxyethylamine

## Conflicts of interest

The authors declare no conflict of interest.

## Supplementary Material
